# Genome-wide analysis of NBS-LRR genes revealed contribution of disease resistance from *Saccharum spontaneum* to modern sugarcane cultivar

**DOI:** 10.3389/fpls.2023.1091567

**Published:** 2023-02-20

**Authors:** Zhengjie Jiang, Mengyu Zhao, Hongzhen Qin, Sicheng Li, Xiping Yang

**Affiliations:** ^1^ State Key Laboratory of Conservation and Utilization of Subtropical Agro-bioresources, Guangxi Key Laboratory of Sugarcane Biology, Guangxi University, Nanning, China; ^2^ National Demonstration Center for Experimental Plant Science Education, College of Agriculture, Guangxi University, Nanning, China

**Keywords:** sugarcane, NBS-LRR genes, phylogenetic analysis, evolutionary analyses, transcriptomic analysis

## Abstract

**Introduction:**

During plant evolution, nucleotide-binding sites (NBS) and leucine-rich repeat (LRR) genes have made significant contributions to plant disease resistance. With many high-quality plant genomes sequenced, identification and comprehensive analyses of NBS-LRR genes at whole genome level are of great importance to understand and utilize them.

**Methods:**

In this study, we identified the NBS-LRR genes of 23 representative species at whole genome level, and researches on NBS-LRR genes of four monocotyledonous grass species, Saccharum spontaneum, Saccharum officinarum, Sorghum bicolor and Miscanthus sinensis, were focused.

**Results and discussion:**

We found that whole genome duplication, gene expansion, and allele loss could be factors affecting the number of NBS-LRR genes in the species, and whole genome duplication is likely to be the main cause of the number of NBS-LRR genes in sugarcane. Meanwhile, we also found a progressive trend of positive selection on NBS-LRR genes. These studies further elucidated the evolutionary pattern of NBS-LRR genes in plants. Transcriptome data from multiple sugarcane diseases revealed that more differentially expressed NBS-LRR genes were derived from S. spontaneum than from S. officinarum in modern sugarcane cultivars, and the proportion was significantly higher than the expected. This finding reveals that S. spontaneum has a greater contribution to disease resistance for modern sugarcane cultivars. In addition, we observed allelespecific expression of seven NBS-LRR genes under leaf scald, and 125 NBS-LRR genes responding to multiple diseases were identified. Finally, we built a plant NBS-LRR gene database to facilitate subsequent analysis and use of NBSLRR genes obtained here. In conclusion, this study complemented and completed the research of plant NBS-LRR genes, and discussed how NBS-LRR genes responding to sugarcane diseases, which provided a guide and genetic resources for further research and utilization of NBS-LRR genes.

## 1 Introduction

Sugarcane (*Saccharum* spp.) is an important sugar economic crop, accounting for 76% of the world’s total sugar production (Zan et al., 2020). Sugarcane cultivation areas are mainly located in tropical and subtropical regions, where high temperature and humidity, and continuous rainy climate make sugarcane be susceptible to various diseases and cause huge economic losses. During long time interactions with pathogens, plants have evolved a well-established immune system during their long evolution to effectively resist pathogenic invasion. Plant immune system includes PAMPs triggered immunity (PTI) induced by pathogen-associated molecular patterns (PAMPs), and effector triggered immunity (ETI) triggered by pathogen effectors (Goff et al., 2002; Jones and Dangl, 2006). PTI is the first level of immune defense for plants, occurring on cell surface, where plants recognize PAMPs through pattern recognition receptors (PRRs) on the cell membrane, and subsequently trigger the immune process. ETI is the second level of defense in plant cells. When some plant pathogen effectors break through the first level of the immune system, they are recognized directly or indirectly by R proteins in plants, which activate downstream signaling pathways and trigger a plant hypersensitivity response (HR) (Martin et al., 2003; Nimchuk et al., 2003) or produce resistance factors to inhibit the spread of pathogens. In recent years, researchers have made great progress in study of plant resistance gene (*R*) in a variety of species, of which Nucleotide Binding Sites-Leucine Rich Repeats (NBS-LRR) is the largest group of *R* genes (Caplan et al., 2008). The protein encoded by NBS-LRR gene has distinct characteristics. Its central structure is a NB-ARC domain, which has the function of molecular switch, and is responsible for the binding and hydrolysis of ATP and GTP (Tameling et al., 2006). The C terminal is a leucine-rich repeat sequence, highly variable, and has the ability to recognize specific pathogens (Meyers et al., 1999). The N-terminus is a variable structure. According to the different domains at the N-terminus, the NBS-LRR gene can be divided into two subfamilies: TIR-NBS-LRR (TNL) and CC-NBS-LRR (CNL). The N-terminus of TNL genes is TIR, while CNL gene is CC (Coiled-Coil) domain (Meyers et al., 1999). In addition to their different domains, they also differ greatly in downstream signaling pathways, indicating that there may be functional differences between the two subfamilies (Tarr and Alexander, 2009). Recently, NBS-LRR gene with RPW8 domain (resistance to powdery mildew 8) is considered as a separate class, the RNL gene, which plays an important role in signaling of disease response (Xiao et al., 2001; Collier et al., 2011; Shao et al., 2016).

Previously, NBS-LRR genes have been identified in various species, including *Arabidopsis thaliana* (Guo et al., 2011), and *Oryza sativa* (Zhou et al., 2004). The results show that the number of NBS-LRR genes in plants is usually in hundreds, reflecting the fact that NBS-LRR genes have an important role in the species. However, the number and characteristics of NBS-LRR genes among species were different. What factors affect the number of NBS-LRR genes in a species? In this study, we identified NBS-LRR at genome-wide level, and performed comparative analysis in 23 representative species. We clarified that species NBS-LRR genes were not related to species genome size or the number of all genes. In addition, we found that whole genome duplication (WGD) and gene expansion affect the number of NBS-LRR genes in species. With the advancement of research, the method of analyzing NBS-LRR genes among multiple species is gradually adopted by researchers (Li et al., 2010). Grass species, *Sorghum bicolor* (*S. bicolor*), *Miscanthus sinensis* (*M. sinensis*) and sugarcane belong to the same monocotyledons (Zhang et al., 2021), and they all have high quality genomes published, which provide a basis for systematic analysis of NBS-LRR genes. In this study, we investigated the sequence characteristics, function, and evolution of conserved NBS-LRR genes in the above grass species to obtain their generality and specificity. Meanwhile, we explored the expression patterns of NBS-LRR genes in responding to diseases and revealed the contribution of *S. spontaneum* to disease resistance in modern sugarcane cultivars using multiple sets of transcriptomic data. These analyses and discoveries complemented the study of the NBS-LRR gene in grass species, and provided guidance and genetic resources for further in-depth studies on the disease resistance mechanism and breeding in sugarcane.

## 2 Methods

### 2.1 Identification of NBS-LRR genes in 23 plant species

Based on the results of Jansen et al. (2007) and Bremer et al. (2009), a total of 23 flowering plants including 19 species with a representative phylogenetic status in taxonomy according to interrelationships of the APG IV orders (Byng et al., 2016) and 4 sugarcane accessions were selected for the study. Among them, there were 13 dicotyledons, and 10 monocotyledons. Their protein sequences as well as genomic information were obtained from Phytozome Plants (https://phytozome-next.jgi.doe.gov/), EnsemblPlants database (http://plants.ensembl.org/species.html), Sugarcane Genome database (http://sugarcane.zhangjisenlab.cn/sgd/html/download.html), the Sugarcane Genome Hub (https://sugarcane-genome.cirad.fr/) and Figshare storage database (https://figshare.com/). Subsequently, protein sequences of the 23 species were annotated using InterProScan 5.48-83.0. Based on the annotation results, the NBS-LRR gene of the species was identified based on the inclusion of NB-ARC and LRR domains. Chloroplast genomic data of the above species were acquired from NCBI, and the species evolutionary tree was constructed using PhyloSuite (Zhang et al., 2020). All of the 63 protein-coding genes (PCGs) shared between 23 species were aligned in batches with MAFFT (v7.313) integrated into PhyloSuite using normal-alignment mode. Maximum likelihood phylogenies were inferred using IQ-TREE (Nguyen et al., 2015) under Edge-unlinked partition model for 50,000 ultrafast (Minh et al., 2013) bootstraps with GTR+F+I+G4, which was the best-fit model according to BIC criterion, as well as the Shimodaira-Hasegawa-like approximate likelihood ratio test (Guindon et al., 2010). The species genome versions, download links, assessment information and chloroplast genome accession numbers were included in **Supplementary Table 1**.

### 2.2 Characterization and analysis of conserved NBS-LRR genes

The MCScanX installed in Tbtools (Wang et al., 2012) was used for rapid identification of intraspecies collinearity NBS-LRR genes with E-value of 10^-5^ in the four closely related monocotyledonous species, *S. bicolor*, *M. sinensis*, *S. spontaneum* and *S. officinarum*. The allelic loss of NBS-LRR gene in *S. spontaneum* and *S.officinarum* was calculated based on their genome annotations (http://sugarcane.zhangjisenlab.cn/sgd/html/download.html). Orthofinder-2.5.4 was used to identify homologous genes between the four species, which were conserved NBS-LRR genes, and the comparison software was Blast (E-value=10^-3)^ (Emms and Kelly, 2015).

### 2.3 Gene composition of conserved NBS-LRR genes

To characterize the NBS-LRR genes, the GC content and CDS length of conserved NBS-LRR gene were evaluated using SeqKit (Shen et al., 2016). Moreover, the characteristics of conserved NBS-LRR gene, including intron size, exon number, and exon size, were estimated based on genome annotations using python script. The statistics of bivariate correlation analysis and analysis of variance (ANOVA) were performed by IBM SPSS Statistics 25.0.

### 2.4 Analysis of motifs, cis-acting and calculation of Ka/Ks ratio

Prediction of conserved motifs of the conserved NBS-LRR genes was performed using the online software MEME (https://meme-suite.org/meme/tools/meme). The top 20 motifs obtained were subjected to functional prediction analysis by Motif Comparison software (https://meme-suite.org/meme/tools/tomtom), and then graphed by ggplot2 R package. The 2000bp sequences before the CDS sequence of conserved NBS-LRR genes were extracted using the Gtf/Gff3 Sequence s Extractor installed in TBtools as the promoter sequences, and submitted to PlantCare (https://bioinformatics.psb.ugent.be/webtools/plantcare/html/) for functional analysis of the cis-acting elements (Lescot, 2002).

Ka/Ks stands for the ratio of non-synonymous to synonymous substitutions, and the Ka/Ks of conserved NBS-LRR genes was calculated based on CDS sequences using the Simple Ka/Ks Calculator installed in TBtools between *S. spontaneum* and *M. sinensis*, *S. bicolor*, *S. officinarum*, respectively.

### 2.5 Transcriptomic analysis

Transcriptomic raw data were downloaded from the ENA database (https://www.ebi.ac.uk/ena/browser/home), and used to analyze the expression of the NBS-LRR gene in sugarcane. A total of 45 RNA-seq datasets were collected for four sugarcane diseases, including sugarcane smut, ratoon stunting, leaf scald, and mosaic virus (**Supplementary Table 2**). Transcriptomic data were uploaded to a local high performance computing server for analysis. Firstly, the transcriptomic sequencing data were quality-controlled using Fastp (Chen et al., 2018). Then clean reads were aligned to transcript sequences of *S. spontaneum* and *S. officinarum* using Bowtie2 (Langmead and Salzberg, 2012). Unique mapped reads were extracted, and quantified using Salmon (Patro et al., 2017). Transcripts per kilobase of exon model per million mapped reads (TPM) was used to estimate the amount of gene expression. In addition, differential expression analysis of between resistant plants and susceptible plants using read counts with the edgeR R package (Robinson et al., 2010). Genes with FDR < 0.05 and |log_2_(fold change)| ≥ 1 estimated by edgeR were assigned as differentially expressed. Replicate-free differential expression analysis was also performed using edgeR on samples between different time points after inoculation (Chen et al., 2008; Anders and Huber, 2010).

### 2.6 Build NBS-LRR gene database

To facilitate the understanding and exploration of the NBS-LRR gene by researchers, we built the NBS-LRR gene database website (http://110.41.19.157:5000/) based on the Linux platform using the Flask framework and SQLite module. A total of 5 HTML pages were built, including Home, Species data, Transcriptomic data, InterProScan, and Blast. User can access the database web page through their browser for corresponding functions.

## 3 Results

### 3.1 Identification of NBS-LRR genes in representative plant species

The two conserved structural domains, NB-ARC and LRR, were used as the basis to identify NBS-LRR genes at genome-wide level in the 23 plant representative specie*s* (**Table 1**). We found that the total number of all genes in a species is positively correlated with genome size (*P <* 0.0001), but the number of NBS-LRR genes were not significantly correlated with genome size and the total number of all genes, showing that the number of NBS-LRR genes was species-specific, and may be the result of species adaptation to their respective ecological environments. This is in line with the findings of Wang et al. (2022b).

**Table 1:** NBS-LRR genes and genome information in the 23 representative plant species.

Species	The number of NBS-LRR genes	The Number of all genes	Genome size (MB)	Ploid
**Blue Star Water Lily** **(*Nymphaea colorata*)**	123	24,059	409	Diploid
**Beet** **(*Beta vulgaris*)**	138	24,255	515.8	Diploid
**Cassava** **(*Manihot esculenta*)**	299	32,805	620.1	Hexaploid
**Cotton** **(*Gossypium hirsutum*)**	493	75,376	2764.8	Tetraploid
**Duckweed** **(*Spirodela polyrhiza*)**	28	19,623	428	Haploid
**Flax** **(*Linum usitatissimum*)**	132	43,471	304.5	Diploid
**Grape** **(*Vitis vinifera*)**	321	31,845	486	Tetraploid
**Mouse-ear cress** **(*Arabidopsis thaliana*)**	138	27,655	115.6	Diploid
**Oil-free camphor** **(*Amborella trichopoda*)**	51	26,846	685	Haploid
**Oilseed rape** **(*Brassica napus*)**	304	85,147	840	Tetraploid
**Peach** **(*Prunus persica*)**	316	26,873	219.6	Diploid
**Red clover** **(*Trifolium pratense*)**	259	39,948	420	Tetraploid
**Rice** **(*Oryza sativa*)**	143	42,189	364.3	Diploid
**Setaria** **(*Setaria viridis*)**	245	38,334	382.1	Diploid
**Sesame** **(*Sesamum indicum*)**	115	27,147	410.3	Haploid
**Silvergrass** **(*Miscanthus sinensis*)**	478	67,789	2007.04	Diploid
**Sorghum** **(*Sorghum bicolor*)**	244	34,129	687	Diploid
**Sunflower** **(*Helianthus annus*)**	258	52,243	3427.2	Diploid
**Sugarcane** **(*R570*)**	60	25,316	407.36	Aneuploid
**Sugarcane** **(*Saccharum spontaneum*)**	468	83,825	2990.08	Octophera
**Sugarcane** **(*Saccharum officinarum*)**	741	117,927	6481.92	Octophera
**Sugarcane** **(*SP80-3280*)**	597	47,496	530.66	Aneuploid
**Tomato** **(*Solanum lycopersicum*)**	156	34,075	950	Diploid

The genome size of plants were estimated based on assembly genome.

**Figure 1:**
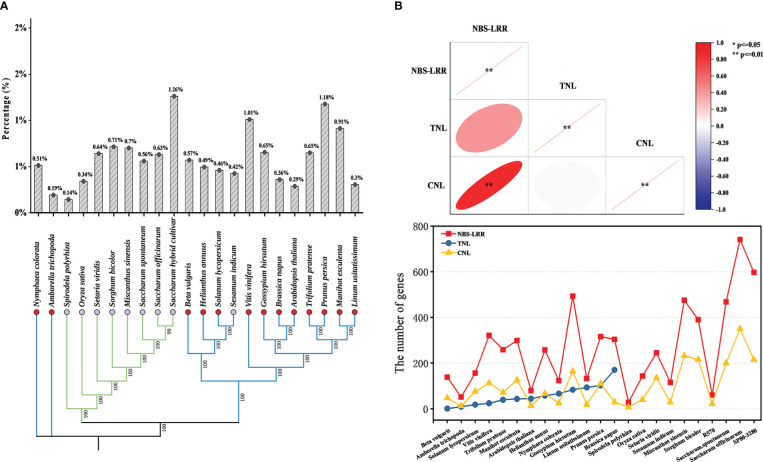
Comparative analysis of NBS-LRR genes in the 23 representative plant species. **(A)** Species phylogenetic relationships and the proportion of the number of NBS-LRR genes to the number of all genes. Blue lines represented dicotyledonous plants, and green lines represented monocotyledonous plants. The red circle showed the species contains “TNL”, while the purple circle showed the species did not contain “TNL”. Percentage represents the proportion of NBS-LRR genes in the total number of genes for each species **(B)** Trends for the number of different types of NBS-LRR genes. Heatmap indicating correlation between different types of genes. The red ellipse means positive correlation and the flatter the ellipse, the smaller the P-value.

To explore the changes in NBS-LRR genes during species evolution, we used the chloroplast genomes to construct a phylogenetic tree and to count the proportion of NBS-LRR genes in species (**Figure 1A**). The number of NBS-LRR genes did not correlate with the evolutionary process of plant species. Analysis of NBS-LRR genes and their subclasses showed that the number of NBS-LRR genes was positively correlated with the number of CNL genes (*P* < 0.0001), but not significantly with TNL genes, which is consistent with the view of Liu et al. (2021) (**Figure 1B**). All dicotyledonous species except *Sesamum indicum* (*S. indicum*) contained TNL and CNL genes, whereas no TNL gene was found in monocotyledonous plants, which is consistent with the results of previous studies (Meyers et al., 1999; Zhou et al., 2004; Gao et al., 2011; Liu et al., 2021). In fact, TIR and CC domains were not always independent, such as an NBS-LRR gene (Tp57577_TGAC_v2_mRNA32981) of Red clover (*Trifolium pratense*) contained both domains according to the results in this study.

### 3.2 Characterization and phylogenetic analysis of NBS-LRR genes in grass species

After removal of alleles of the same gene, 299, 340, 244 and 478 NBS-LRR genes were identified in *S. spontaneum*, *S. officinarum*, *S. bicolor* and *M. sinensis*, with 132 (44%), 157 (46.2%), 135 (55.3%) and 232 (48.5%) CNL genes, respectively. Gene expansion or loss may lead to the differences in the number of NBS-LRR genes among species. We first explored the collinearity relationships of the NBS-LRR gene within species using MCScanX for identification of duplicated NBS-LRR genes (**Supplementary Figure 1**). After removing alleles, 18 gene pairs formed by 36 NBS-LRR genes were identified in *S. spontaneum*, of which 18 genes (50%) were located on Chr2 (8 genes) and Chr5 (10 genes). A total of 47 gene pairs formed by 75 genes were identified in *S. officinarum*, with 41 genes (54.7%) on Chr5. Both *M. sinensis* and *S. bicolor* were diploids, but a large difference in the number of NBS-LRR dulicated gene pairs between them. In *M. sinensis*, 51 gene pairs of NBS-LRR genes formed by 93 genes were found, with the largest number of genes on Chr9 (12 genes). Only two NBS-LRR gene pairs were found in the *S. bicolor*, distributed on Chr3 and Chr5, respectively. *M. sinensis* went through recent whole genome duplication (WGD) and chromosome rearrangement events, and its 18 basic chromosomes showed a good syntenic relationship between each other (Cheng et al., 2018), which may cause high duplicated genes identified in *M. sinensis*. In addition, we estimated the retention of alleles of the NBS-LRR genes within in *S. spontaneum* and *S. officinarum*, and the results showed that loss of a large number of alleles occurred in sugarcane. In the tetraploid *S. spontaneum* genome, only 18 NBS-LRR genes (6%) retained 4 alleles, 281 NBS-LRR genes (94%) lost 1 to 3 alleles, of which 46.6% were CNL genes and 53.4% were truncated CNL genes lacking the CC structure. In the octaploid *S. officinarum* genome, only 3 NBS-LRR genes (1.3%) retained 8 alleles, and 337 NBS-LRR genes (98.7%) lost 1 to 7 alleles, with CNL genes accounting for 46.3% and truncated CNL genes for 53.7%. CNL genes lost fewer alleles than truncated CNL genes (*P* < 0.001), indicating that CNL genes were likely more conserved in polyploid sugarcane genome evolution.

In order to better investigate the functional and evolutionary relationships of NBS-LRR genes in sugarcane, OrthoFinder was used to analyze homologous NBS-LRR genes called conserved NBS-LRR genes in four monocotyledonous grass species, including *S. spontaneum*, *S. officinarum*, *S. bicolor* and *M. sinensis* (**Table 2**). In total, 166, 121, 125 and 181 conserved NBS-LRR genes were identified in *S. spontaneum*, *S. officinarum*, *S. bicolor* and *M. sinensis*, accounting for 35.5%, 16.7%, 51.2%, and 37.9% of the number of NBS-LRR genes in the four species, and there were 75 (45.2%), 62 (51.2%), 65 (52%) and 99 (54.7%) CNL genes in the conserved NBS-LRR genes, respectively. We found that the proportion of CNL genes in the total number of NBS-LRR genes was higher in *S. bicolor* than in other three species, while the proportion of CNL genes in conserved NBS-LRR genes was increased compared to the proportion of CNL at the genome-wide level. The proportion of CNL genes in conserved genes was higher in *S. bicolor* and *M. sinensis* than in sugarcane.

**Table 2:** Summary of NBS-LRR genes in the four grass species.

Species	NBS-LRR genes		^a^ NBS-LRR genes (Conserved)	
	NBS-LRR	^b^CNL (%)	NBS-LRR (%)	^c^CNL (%)
*S. spontaneum*	299	132 (44%)	166 (35.5%)	75 (45.2%)
*S. officinarum*	340	157 (46.2%)	121 (16.7%)	62(51.2%)
*S. bicolor*	244	135 (55.3%)	125 (51.2%)	65 (52%)
*M. sinensis*	478	232 (48.5%)	181 (37.9%)	99 (54.7%)

a Conserved NBS-LRR genes among the four species were characterized by OrthoFinder.

b The percentage was calculated as the number of CNL genes or conserved genes to the number of NBS-LRR genes.

c The percentage was calculated as the number of conserved CNL genes to the number of conserved NBS-LRR genes.

### 3.3 Gene composition and evolutionary analyses of conversed NBS-LRR genes

For investigating the structure of the NBS-LRR gene, we compared the GC content, CDS length, introns and exons of the conserved NBS-LRR gene in the four monocotyledonous plants. The average GC content of the conserved NBS-LRR gene was approximately 45% with no significant differences among the four species (**Supplementary Figure 2B**). The mean GC content of CNL genes was lower than that of truncated CNL genes in all four species (*P* < 0.05) (**Supplementary Figure 2C**). The CDS length of the NBS-LRR gene differed among the four species, with *S. bicolor* having the largest mean CDS and *S. officinarum* having the smallest, at 3,352.6 bp and 3,035.6 bp, respectively (*P* < 0.001). Except for *S. bicolor*, the mean CDS length of CNL genes was lower than that of truncated CNL genes in the other three monocotyledons (*P* < 0.001) (**Supplementary Figure 3C**).

There were also significant differences in the intron size of NBS-LRR gene among the four species (*P* < 0.01), among which the intron length of *S. officinarum* was the largest and that of *S. bicolor* was the smallest (**Supplementary Figure 4A**). By analyzing their exons, it was found that *M. sinensis* had the largest number of exons and *S. officinarum* had the least (*P* < 0.01) (**Supplementary Figure 4B**). The exon fragment length of NBS-LRR genes in sorghum was the longest and the shortest in *S. spontaneum* (*P* < 0.01) (**Supplementary Figure 4C**). The intron fragment length of CNL genes was significantly smaller than that of truncated CNL genes (*P* < 0.01) (**Supplementary Figure 5A**). The mean exon length of CNL genes was greater than that of truncated CNL genes except for *S. spontaneum* (*P* < 0.05) (**Supplementary Figure 5C**).

**Figure 2:**
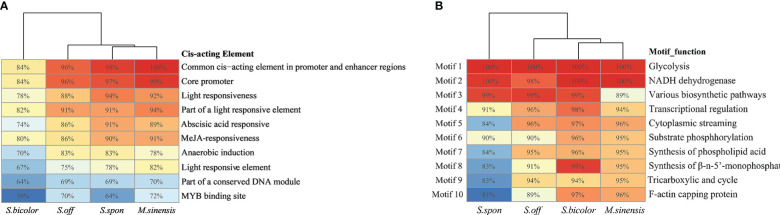
Analysis of motifs and cis-acting elements of conserved NBS-LRR genes in the four grass species. *Saccharum spontaneum* (*S.spon*); *Saccharum officinarum* (*S.off*); *Sorghum bicolor* (*S.bicolor*); *Miscanthus sinensis* (*M. sinensis*). The numbers in heatmap represent the percentages of genes containing the element/Motif of conserved NBS-LRR genes. **(A)** Analysis of cis-acting elements. **(B)** Analysis of protein motifs.

Analysis of cis-acting elements and protein motifs was useful for exploring the function of NBS-LRR genes. The functions of the predicted cis-acting elements were primarily related to light response, phytohormones response, stress response, and plant growth and metabolism. The most widely distributed top 10 cis-acting elements were mainly involved in the regulation of transcription, response to light, induction of anaerobic motility, and metabolism of gibberellin and methyl jasmonate (**Figure 2A**), while methyl jasmonate played an important role in disease resistance in plants (Pichersky and Gershenzon, 2002). In addition, we also found that NBS-LRR genes in *S. bicolor* contained the fewest cis-acting elements among the four grass species (*P* < 0.05). The most distributed top 10 protein motifs in the NBS-LRR genes of the four grass species were analyzed, and their functions were primarily related to catalyzing the reversible interconversion of 3-phosphoglycerate and dihydroxyacetone phosphate, catalytic substrate phosphorylation, transcriptional regulation, and synthesis pathways of various biological substances (**Figure 2B**). Based on protein motif, *M. sinensis* were clustered close to that of Saccharum genus (*S. spontaneum* and *S. officinarum*), while *M. sinensis* and *S. bicolor* were in the same clusters based on promoter elements, illustrating differences in the evolution of the conserved NBS-LRR genes between species in terms of regulatory elements and functional motifs.

**Figure 3:**
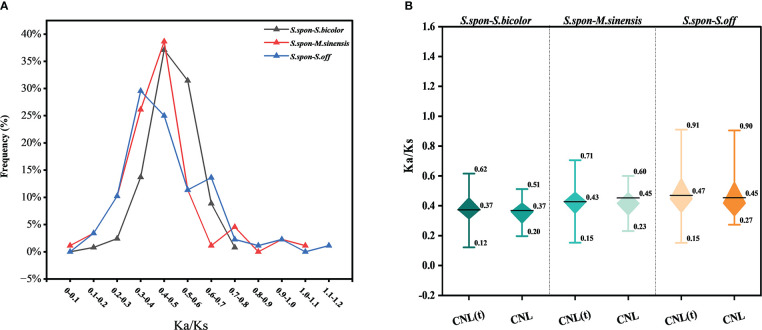
The ratio of nonsynonymous (Ka) to synonymous (Ks) nucleotide substitution rates of conserved NBS-LRR genes between the four grass species. *Saccharum spontaneum* (*S.spon*); *Saccharum officinarum* (*S.off*); *Sorghum bicolor* (*S.bicolor*); *Miscanthus sinensis* (*M. sinensis*). **(A)** Distribution interval of Ka/Ks values of conserved NBS-LRR genes. The percentage indicates the proportion of homologous gene pairs in that Ka/Ks interval in the total number of homologous gene pairs. **(B)** Comparison of Ka/Ks values of truncated CNL genes and CNL genes. The black line in the box represents the average value of the data. CNL(t) stands for truncated CNL gene.

The ratio of nonsynonymous (Ka) to synonymous (Ks) nucleotide substitution rates for conserved NBS-LRR genes among grass species showed that the Ka/Ks gene frequencies were different between species (**Figure 3A**). A total of 88 shared gene pairs were used for calculation of Ka/Ks values among comparisons between *S. spontaneum* and *M. sinensis*, *S. bicolor*, *S. officinarum*, respectively. The proportion of gene pairs with high Ka/Ks values (Ka/Ks > 0.7) was gradually increasing in *S. spontaneum* compared with *S. bicolor*, *M. sinensis* and *S. officinarum* repectively, and the Ka/Ks value of one pair of homologous gene pairs between *S. spontaneum* and *S. officinarum* was above 1.1 (*Sspon.07G0017980-1A* and *Soff.08G0002710-1A*), indicating that these NBS-LRR genes were subject to positive selection during species evolution. In addition, Ka/Ks ratios of *Sspon.07G0017980-1A* with *Sobic.003G317300* in *S. bicolor* and *Misin05G292700* in *M. sinensis* respectively were greater than 0.7. The Ka/Ks values of CNL and truncated CNL genes confirmed the above trends (**Figure 3B**), but not significant differences were observed between the two subfamilies.

### 3.4 Transcriptomic analysis of NBS-LRR genes in sugarcane

**Figure 4:**
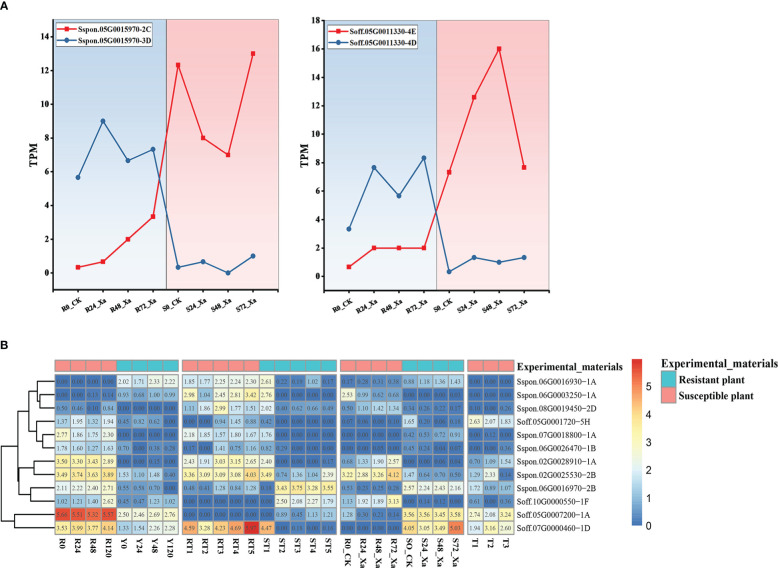
Transcriptomic analysis in multiple RNA-seq datasets. **(A)** Differential expression analysis of *S. spontaneum* and *S. officinarum* alleles. **(B)** Differentially expressed NBS-LRR genes in multiple diseases.

NBS-LRR genes, as one of the most important disease resistance genes in plants, play an important role in the resistance to pathogens (DeYoung and Innes, 2006). We analyzed transcriptomic data related to sugarcane smut, ratoon stunting, leaf scald, and mosaic virus diseases. After filtering out low-quality reads, the clean reads had a Q20 over 90% and a GC content of 53.65%~ 54% (**Supplementary Table 3**), showing a high quality of the sequencing data. Considering alleles of the same genes, 131, 126 and 269 differentially expressed NBS-LRR genes were identified between resistant and susceptible plants after being challenged by pathogens of sugarcane smut, ratoon stunting, and leaf scald, respectively, and 18 differentially expressed NBS-LRR genes were identified between infected and healthy sugarcane for mosaic virus disease. After removing alleles based on genome annotations, there were 125, 121, 226, and 18 differentially expressed genes in the four diseases, respectively. Interestingly, the expression patterns among alleles of the same NBS-LRR genes were not always the same. For example, in leaf scald, the expression of the *Sspon.05G0015970-2C* was significantly up-regulated in susceptible plants, while the allele *Sspon.05G0015970-3D* was significantly up-regulated in the resistant plants (**Figure 4A**). A similar situation was observed in the NBS-LRR gene from *S. officinarum*. In leaf scald, *Soff.05G0011330-4E* was significantly up-regulated in resistant plants, while the allele *Soff.05G0011330-3D* in susceptible plants was significantly up-regulated (**Figure 4A**). In this study, we found that, in addition to mosaic virus disease, 6, 5 and 38 genes had alleles in the differentially expressed genes of the three diseases of sugarcane smut, ratoon stunting and leaf scald, respectively, of which only 7 genes of leaf scald had allele-specific expression.

After removal of alleles, among the differentially expressed NBS-LRR genes for sugarcane smut, the number of genes from *S. spontaneum* and *S. officinarum* was 62 (49.6%) and 63 (50.4%), respectively, of which 28 genes were up-regulated from *S. spontaneum* and 31 genes were up-regulated from *S. officinarum*. Among the differentially expressed NBS-LRR genes for ratoon stunting disease, the number of genes from *S. spontaneum* and *S. officinarum* were 70 (56.9%) and 51 (43.1%), respectively, of which 36 genes were up-regulated from *S. spontaneum* and 22 genes were up-regulated from *S. officinarum*. Among the differentially expressed NBS-LRR genes for leaf scald, the number of genes from *S. spontaneum* and *S. officinarum* were 127 (55.3%) and 99 (44.7%), respectively, of which 54 genes were up-regulated from *S. spontaneum* and 53 genes from *S. officinarum*. Among the differentially expressed NBS-LRR genes for mosaic virus disease, the number of genes from *S. spontaneum* and *S. officinarum* were nine for each species, of which six were upregulated in the differentially expressed genes from *S. spontaneum* and four from *S. officinarum*. In modern sugarcane cultivars, about 10-15% of the genome is derived from *S. spontaneum* and 80% from *S. officinarum* (Zhang et al., 2018). The proportion of differentially expressed genes from *S. spontaneum* in sugarcane cultivars was much higher than the theoretical value (*P* < 0.001, based on *S. spontaneum* contributing to 20% genomes in sugarcane cultivars), indicating that *S. spontaneum* has a greater contribution to disease resistance for modern sugarcane cultivars.

We also investigated differential expressed genes for each disease at different time points after inoculation (**Supplementary Figure 6**). In sugarcane smut, three NBS-LRR genes from *S. officinarum* were differentially expressed at 24 hours after inoculation. In contrast, NBS-LRR genes from *S. spontaneum* were upregulated at 48 hours after inoculation in the resistant plants, and at 120 hours in susceptible plants. The NBS-LRR genes seems to have different patterns after pathogen challenging, and the genes from *S. officinarum* first responded sugarcane smut. For Ratoon stunting disease, the trend of NBS-LRR gene expression pattern from *S. spontaneum* and *S. officinarum* was the same. For Leaf scald, the number of differentially up-regulated NBS-LRR genes from *S. spontaneum* and *S.officinarum* in resistant plant continued to increase after inoculation, while in susceptible plant the number of differentially up-regulated NBS-LRR genes showed a trend of first decreasing and then increasing patterns. Comparing healthy and susceptible sugarcane challenged by mosaic virus, we found that the number of differential up-regulation of NBS-LRR genes were higher than the differential down-regulation genes from *S. spontaneum*, while the expression pattern was opposite for *S. officinarum*.

Integration of the four transcriptomic data showed that 125 NBS-LRR genes were differentially expressed in at least two diseases. Here, we screened a total of 12 genes that were significantly differentially expressed (FDR < 0.05 and |log_2_(fold change)| ≥ 2) in common among sugarcane smut, ratoon stunting, and leaf scald disease, two of which were differentially expressed in all four diseases, *Sspon.02G0025530-2B* and *Soff.05G0001720-5H* (**Figure 4B**). Some genes were able to respond to multiple diseases. For example, regarding ratoon stunting, and leaf scald, the expression of *Sspon.06G0016970-2B* in resistant plants was significantly up-regulated after inoculation. For sugarcane smut, ratoon stunting and leaf scald disease, the expression of *Sspon.02G0025530-2B* in susceptible plants was significantly up-regulated after inoculation. In particular, the gene *Sspon.06G0016970-2B*, which encodes the RGA5 disease resistance protein in rice, is tightly linked to RGA4 in an inverted tandem fashion at the Pi-CO39/Pia resistance locus, and ectopic activation of RGA4/RGA5 has been reported to confer resistance to bacterial wilt and bacterial leaf streak (Hutin et al., 2016).

### 3.5 Construction of NBS-LRR gene database

For researchers to quickly access information on the plant NBS-LRR genes, we constructed the NBS-LRR gene database (http://110.41.19.157:5000/) using Argon Design (**Supplementary Figure 7A**). This database consisted of two modules, data and tools. The data module was composed of species information including the genome size, ploid, number of all genes, and number of NBS-LRR genes identified (**Supplementary Figure 7B**), and transcriptomic data including results obtained in this study (**Supplementary Figure 7C**). The Tools module contained Blast and InterProScan tools, which allow users to search our database and do protein annotation using their sequences of interest (**Supplementary Figures 7D, E**). Compared with other similar databases, such as RPGdp (http://www.prgdb.org/prgdb/), DeepLRR (http://lifenglab.hzau.edu.cn/DeepLRR/index.html), and etc., our database provided comprehensive information on NBS-LRR genes in sugarcane for the first time, especially adding information on the expression of NBS-LRR genes under several sugarcane disease stresses, which facilitated users in-depth study on corresponding area.

## 4 Discussion

### 4.1 NBS-LRR genes are complex and variable in species evolutionary

Since Johal and Briggs isolated the first plant *R* gene, *Hm1*, from maize (*Zea mays L*) in 1992 (Johal and Briggs, 1992), researchers have identified many *R* genes in a variety of plants, of which more than 70% are classified in NBS-LRR class (McHale et al., 2006). Ancient origin and large number of subfamilies of plant NBS-LRR genes have certainly made it more difficult to explore the evolutionary patterns of them among species. In our study, NBS-LRR genes were identified in 23 representative plants, and their comparisons showed that the number of NBS-LRR genes was independent of species divergence and genome size, and the percentage of the number of NBS-LRR genes to the total number of genes differed between species even between the same clade and genus. WGD may be one of the major reasons affecting the number of NBS-LRR genes among species. Both WGD and large-scale duplication of chromosomal segments lead to genome duplications within a species (Zhang, 2003), resulting in expansion of alleles of the same genes in the same species. Based on the annotation results of Interproscan, 468 and 741 NBS - LRR genes were annotated in *S. spontaneum*, *S. officinarum*, respectively, where the number of alleles of other genes was 169 and 401, respectively. The large number of gene expansion were likely related to the fact that sugarcane has experienced at least two WGD events in its evolutionary history (Zhang et al., 2018). Gene duplication also affects the number of species NBS-LRR genes. After removal of alleles of the same gene, a total of 36, 75, 93 and 4 NBS-LRR genes were identified in *S. spontaneum*, *S. officinarum*, *M. sinensis* and *S. bicolor*, respectively, forming 18, 47, 51 and 2 duplicated gene pairs, which were derived from segmental duplication. We evaluated the effects of two factors on the number of NBS-LRR genes in *S. spontaneum* and *S. officinarum*. At least 36.1% and 45.9% of NBS-LRR genes in *S. spontaneum* and *S. officinarum*, respectively, were generated by the effects of WGD, while 18 (3.8%) and 47 (6.3%) of NBS-LRR genes, respectively, were generated by gene expansion. Based on this, we speculate that WGD may have a greater effect on the number of NBS-LRR genes in sugarcane compared to gene expansion (*P* < 0.05). In contrast to gene duplication, gene loss is another reason to change the number of genes. For instance, *A. thaliana*, which has undergone multiple WGD events in its evolutionary history (Blanc et al., 2003; Bowers et al., 2003), still has a genome size of ~150 MB, and the latest high-quality *A. thaliana* genome assembled by Wang et al. (2022a) was only about 133 Mb, implying that the vast majority of duplicated genes are not retained after a polyploidization events. In fact, gene loss was an inevitable trend of genome reconstruction after polyploidization (Lynch and Conery, 2000; Liang and Schnable, 2018). In *S. spontaneum* and *S. officinarum*, more than 94% of NBS-LRR genes lost at least one alleles. Balance of energy costs may be another reason to maintain a relative small number of NBS-LRR genes in species (Tian et al., 2003). Plants did not expand NBS-LRR genes blindly, and their number remained below 1.5% of the total number of genes in the species (**Figure 1A**). It has been reported that plants loses some of its *R* genes to avoid wasting costs (Brown, 2002).

The differentiation and evolution patterns of different subfamilies of NBS-LRR genes are also long-standing puzzles for researchers. TNL and CNL genes are the two major subfamilies of NBS-LRR genes. The origin time of NBS-LRR gene has been proven to be earlier than the separation of chloroplast and streptococcus (Shao et al., 2019), and (Shao et al., 2016) found that the differentiation time of TNL subfamily genes may be earlier than CNL genes. Among the 23 species we studied, TNL and CNL genes were found in the earliest differentiated angiosperms, *Amborella trichopoda*, *Nymphaea colorata* and *Nymphaea versipellis*. As species evolved, TNL genes only existed in dicotyledons, but not in monocotyledons. It is still unclear that why TNL genes are lost in monocotyledons. In addition, according to our study, not all dicotyledonous plants contained the TNL genes. For example, *S. indicum* is a dicotyledonous plant, but did not contain the TNL genes. In addition, the number of CNL genes was positively correlated with the number of NBS-LRR genes, while the number of TNL genes not. The proportion of CNL genes in *S. bicolor* and *M. sinensis* was higher than that in sugarcane. In the other three monocots except sorghum, the proportion of CNL genes in the conserved NBS-LRR gene was higher than that of the whole genome level. Moreover, the statistical analyses of allele loss in *S. spontaneum* and *S. officinarum* showed that CNL genes was more difficult to lost alleles than truncated CNL genes. The results supported that TNL and CNL genes were likely to have different evolutionary patterns.

### 4.2 NBS-LRR genes in modern sugarcane cultivar

In the breeding process of modern sugarcane cultivar, *S. officinarum* was crossed with *S. spontaneum*, and the offspring obtained was backcrossed with *S. officinarum* for several generations to obtain cultivars with high sugar and high stress resistance (Zhang et al., 2021). In this study, we analyzed multiple sets of transcriptomic data of sugarcane challenged by sugarcane diseases. It was surprising that the differentially expressed genes from *S. spontaneum* in modern sugarcane cultivar were more than those from *S. officinarum*, and the proportions of differentially expressed NBS-LRR from *S. spontaneum* were significantly higher than the expected. This result indicated that the NBS-LRR genes from *S. spontaneum* were selected in sugarcane breeding program, and confirmed its significant contribution to disease resistance of modern sugarcane cultivars although *S. spontaneum* contributes to less than 20% genomes of sugarcane cultivars.

Plant *R* genes usually target specific pathogen gene in defense against pathogens, but some *R* genes can mediate defense against multiple diseases. These genes were called multi-disease resistance genes (Fukuoka et al., 2014). For example, *Lr34* and *Lr67* genes have been shown to be resistant to a variety of pathogens in wheat including *Puccinia triticina*, *Puccinia striiformis* and *Blumeria graminis* (Krattinger et al., 2009; Moore et al., 2015). In this study, some NBS-LRR genes, such as *Sspon.02G0025530-2B* and *Soff.05G0001720-5H*, were also found to respond to various sugarcane diseases. The mechanisms of NBS-LRR genes responding to multiple diseases are inconsistent and complex. Studies have shown that *Lr34*-encoded protein is located on the cell membrane, which may affect the cell membrane structure by regulating phospholipid metabolism, and mediate abscisic acid (ABA) signaling pathway to achieve resistance to multiple diseases (Deppe et al., 2018; Krattinger et al., 2019). *lr67* encodes a hexose transporter that may be related to glucose metabolism in mediating disease resistance (Milne et al., 2019). NBS-LRR genes stimulate strong ETI immune response by directly or indirectly identifying pathogen effector, resulting in an allergic reaction characterized by programmed cell death to resist the invasion of the pathogen (Jones and Dangl, 2006). We speculated that NBS-LRR genes responding to a variety of sugarcane diseases may be due to their ability to identify core effectors shared by pathogens. However, molecular mechanisms on how these genes work needed further investigation.

Allele specific expression (ASE) of NBS-LRR gene exists in sugarcane under disease stress. It was reported that ASE is a critical gene regulation, and the influence of cis-acting genetic variation is one of the main reasons for the specific expression between alleles (Gaur et al., 2013; Hill et al., 2021). ASE were ubiquitous in a variety of organisms (Knight, 2004), and studies have shown that ASE plays an important role in *Zea mays* (Springer and Stupar, 2007), *A. thaliana* (Todesco et al., 2010), and *Oryza sativa* (He et al., 2010). In addition, studies on human diseases have shown that ASE of genes encoding pathogenic enzymes can affect an individual ‘s susceptibility to disease(Emison et al., 2010; Finch et al., 2011; Berlivet et al., 2012; EMBRACE et al., 2012). Among the differentially expressed genes in response to leaf scald, we identified seven NBS-LRR genes with specific expression profiles, accounting for 3% of the differentially expressed genes in leaf scald, and, these NBS-LRR genes encompassed both *S. spontaneum* and *S. officinarum* sources, suggesting that ASE was also an important regulation mechanism in sugarcane disease resistance. However due to the complexity of ASE regulation mechanism and the limitation of detection technology, the research on ASE is still in its infancy, and needs further exploration.

## 5 Conclusion

By identification of genome-wide NBS-LRR genes in 23 representative species, and comparisons in four grass species, we found that the number of NBS-LRR genes did not correlate with their genome size, and total number of genes, and whole genome duplication may be the main factor affecting the number of NBS-LRR genes in sugarcane. In addition, our comparisons supported the previous researchers’ view that TNL and CNL had different evolutionary patterns. Transcriptomic analysis of sugarcane challenged by different diseases showed that more differentially expressed NBS-LRR genes were derived from *S. spontaneum* than from *S. officinarum*, and the proportion of differentially expressed genes from *S. spontaneum* was significantly higher than the expected ratio in modern sugarcane cultivars, revealing its contribution of disease resistance. Moreover, allele specific expression of NBS-LRR genes were observed in responding to pathogen infection in sugarcane. In conclusion, the comprehensive analyses of plant NBS-LRR genes provided a deeper exploration of the evolutionary patterns of NBS-LRR genes, and contributed important gene resources to sugarcane improvement on disease resistance.

## Data availability statement

The original contributions presented in the study are included in the article/**Supplementary Material**. Further inquiries can be directed to the corresponding author.

## Author contributions

ZJ and XP conceived and designed the project. ZJ and MY obtained and analyzed the data and wrote the manuscript. SC participated in the data analysis and discussion. HZ was involved in building the plant NBS-LRR gene database. XP revised the manuscript. All authors contributed to the article and approved the submitted version.
