# Supplementary material for: Genome-wide analysis of NBS-LRR genes revealed contribution of disease resistance from Saccharum spontaneum to modern sugarcane cultivar
Source: Front Plant Sci. 2023 Feb 20;14:1091567. doi: 10.3389/fpls.2023.1091567 (PMC9986449; doi:10.3389/fpls.2023.1091567)
Supplement: Supplementary file 1 [file DataSheet_1.docx]

Supplementary Material

# Supplementary Figures and Tables

## Supplementary Figures


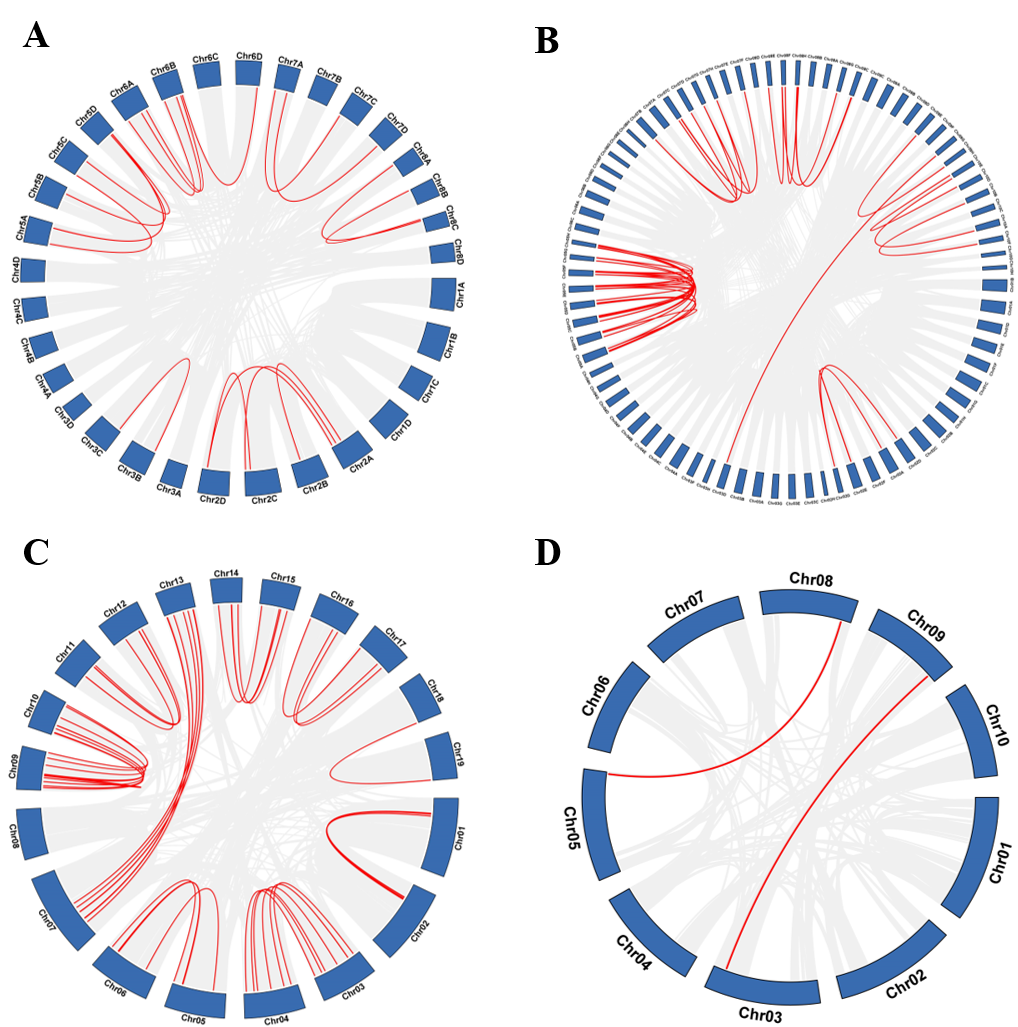


**Supplementary Figure 1** The Collinearity analysis of NBS-LRR genes (with alleles removed) in four monocotyledonous grass species. The red line represents the presence of co-collinearity between genes. **(A)** *S. spontaneum*. **(B)** *S. officinarum*. **(C)** *M. sinensis*. **(D)** *S. bicolor.*

**Supplementary Figure 2** GC content analysis of NBS-LRR genes in four grass species. The upper and lower lines on the outside of the box represent the upper and lower limits of the data, and the black line in the box represents the average value of the data. *Saccharum spontaneum* (*S.spon*); *Saccharum officinarum* (*S.off*); *Sorghum bicolor* (*S.bicolor*); *Miscanthus sinensis* (*M.sinensis*). **(A)** The GC content of all NBS-LRR genes after removal of alleles. **(B)** The GC content of NBS-LRR homologous. **(C)** The GC content of truncated CNL genes and CNL genes in NBS-LRR homologous. CNL(t) stands for truncated CNL gene; CNL (CC-NBS-LRR). The upper and lower black lines on the outside of the box represent the upper and lower limits of the data, and the black line in the box represents the average value of the data.

**Supplementary Figure 3** CDS length analysis of NBS-LRR genes in four grass species. The upper and lower lines on the outside of the box represent the upper and lower limits of the data, and the black line in the box represents the average value of the data. *Saccharum spontaneum* (*S.spon*); *Saccharum officinarum* (*S.off*); *Sorghum bicolor* (*S.bicolor*); *Miscanthus sinensis* (*M.sinensis*). **(A)** The CDS length of all NBS-LRR genes after removal of alleles. **(B)** The CDS length of NBS-LRR homologous. **(C)** The CDS length of truncated CNL genes and CNL genes in NBS-LRR homologous. CNL(t) stands for truncated CNL gene; CNL (CC-NBS-LRR).


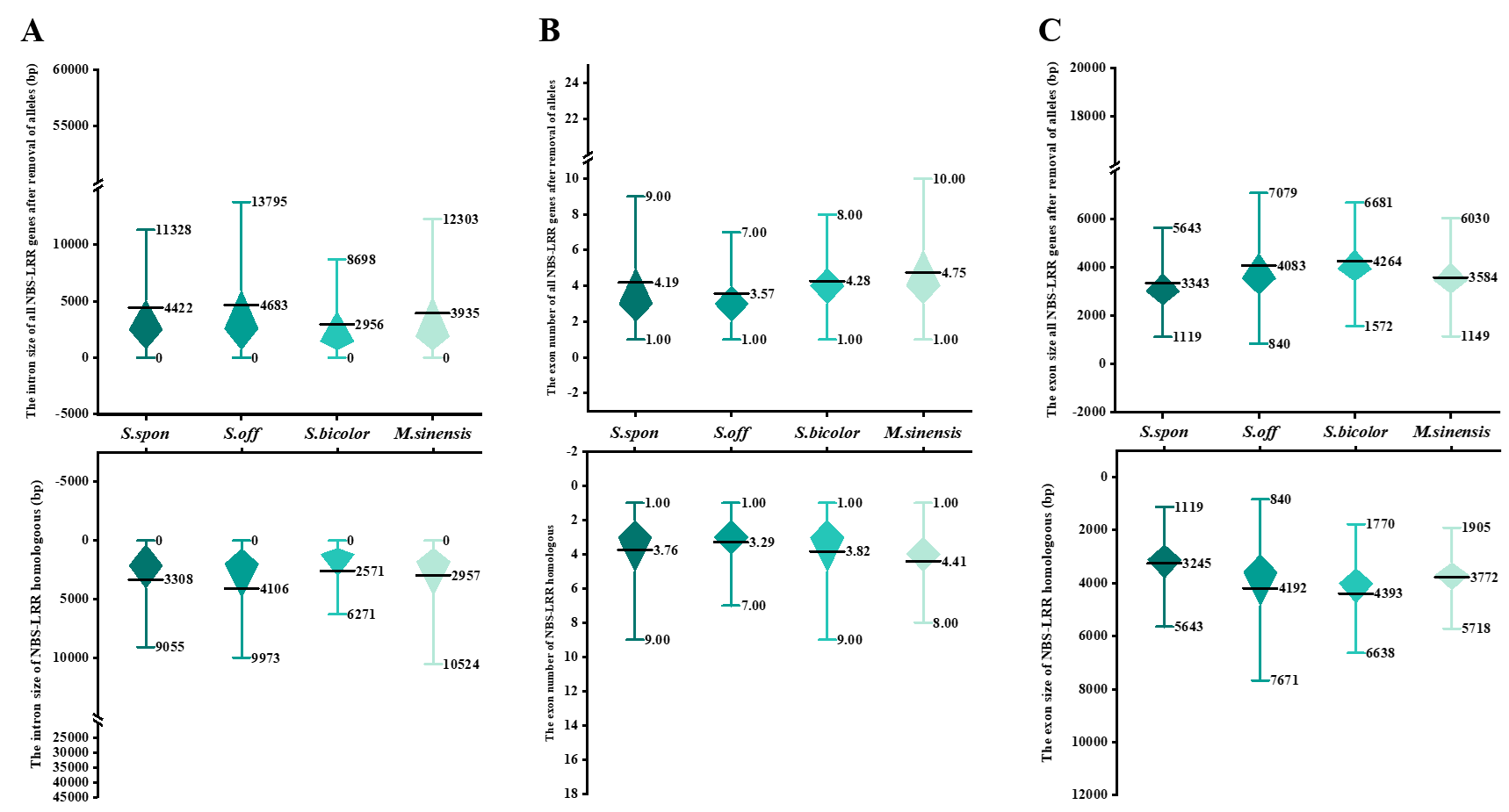


**Supplementary Figure 4** Analysis of intron size, exon number and exon size. The upper and lower lines on the outside of the box represent the upper and lower limits of the data, and the black line in the box represents the average value of the data. *Saccharum spontaneum* (*S.spon*); *Saccharum officinarum* (*S.off*); *Sorghum bicolor* (*S.bicolor*); *Miscanthus sinensis* (*M.sinensis*). **(A)** The Intron size analysis of *NBS-LRR* genes in four grass species. **(B)** The exon number analysis of *NBS-LRR* genes in four grass species. **(C)** The exon size analysis of *NBS-LRR* genes in four grass species.

**Supplementary Figure 5** Analysis of intron size, exon number and exon size of CNL and truncated CNL genes. The black line in the box represents the average value of the data. *Saccharum spontaneum* (*S.spon*); *Saccharum officinarum* (*S.off*); *Sorghum bicolor* (*S.bicolor*); *Miscanthus sinensis* (*M. sinensis*). CNL(t) stands for truncated CNL gene; CNL (CC-NBS-LRR). **(A)**The Intron size analysis of CNL and truncated CNL genes from NBS-LRR homologous in four species. **(B)** The exon number analysis of CNL and truncated CNL genes from NBS-LRR homologous in four species. **(C)** The exon size analysis of CNL and truncated CNL genes from NBS-LRR homologous in four species.


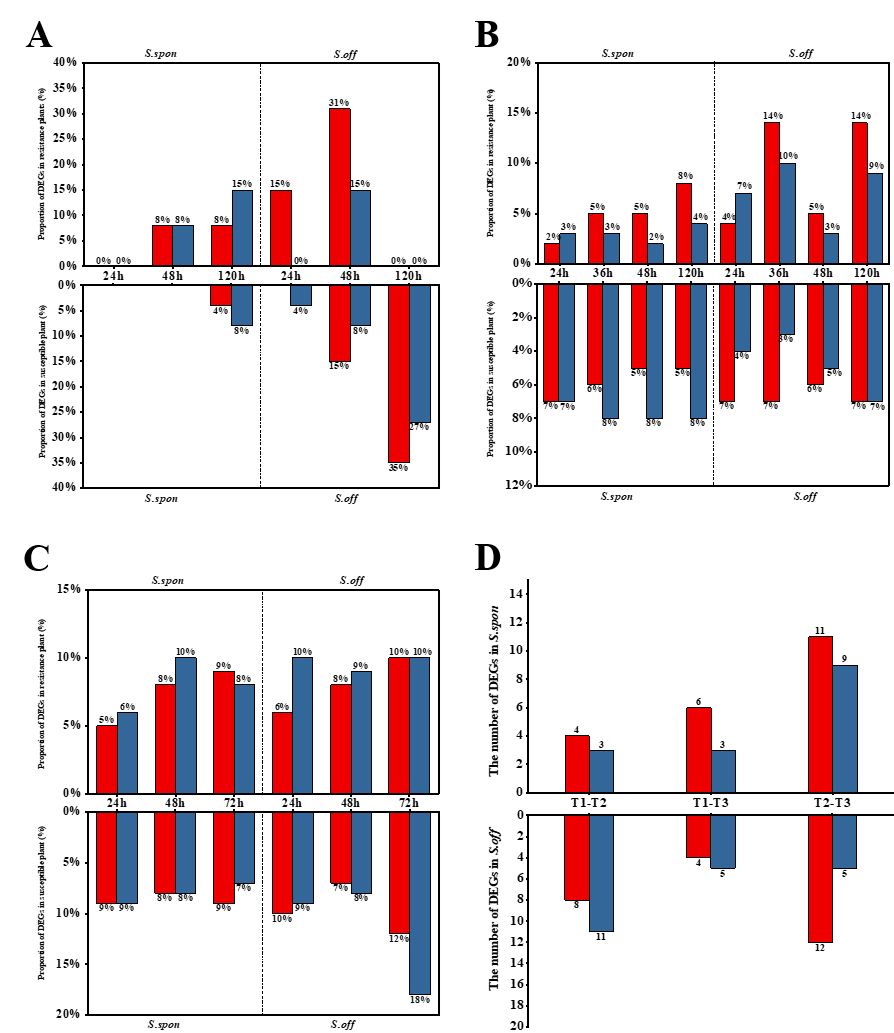


**Supplementary Figure 6** Number of differentially expressed genes at different time points after inoculation with pathogens. The proportional value represents the number of differential genes at each time point after treatment as a proportion of the total number of differential genes after treatment (*S. spontaneum* & *S. officinarum*).Red shapes represent up-regulated genes, blue shapes represent down-regulated genes *Saccharum spontaneum* (*S.spon*); *Saccharum officinarum* (*S.off*). **(A)** Sugarcane smut. **(B)** Ratoon stunying disease. **(C)** Leaf scald. **(D)** Mosaic virus disease.

**Supplementary Figure.7** The plant NBS-LRR gene database. **(A)** Website Home. **(B)** Data of the species NBS-LRR gene. **(C)** Sugarcane disease transcriptome data. **(D)** Blast. **(E)** InterProScan.

## Supplementary Table

**Supplementary Table 1** The genome versions, download links, assessment information and chloroplast genome accession numbers.

| **Species** | **Genome version** | **Download Links** | **Percentage of Eukaryote BUSCO Genes（%）** | **Contig N50^a^(bp)** | **accession numbers of chloroplast genomes** |
| --- | --- | --- | --- | --- | --- |
| Blue Star Water Lily (*Nymphaea colorata*) | Nymphaea colorata v1.2 | https://data.jgi.doe.gov/refine-download/phytozome?organism=Ncolorata&expanded=566 | 98 | 2,079,871 | XR_004174351 |
| Beet (*Beta vulgaris*) | Beta vulgaris EL10_1.0 | https://data.jgi.doe.gov/refine-download/phytozome?organism=Bvulgaris&expanded=548 | 96.7 | 2,700,559 | DQ073528 |
| Cassava (*Manihot esculenta*) | Manihot esculenta v8.1 | https://data.jgi.doe.gov/refine-download/phytozome?organism=Mesculenta&expanded=671 | 98.3 | 3,258,867 | NC_035161 |
| Cotton (*Gossypium hirsutum*) | Gossypium hirsutum v3.1 | https://data.jgi.doe.gov/refine-download/phytozome?organism=Ghirsutum&expanded=578 | 99 | 39,954,488 | MW843817 |
| Duckweed （*Spirodela polyrhiza*） | Spirodela polyrhiza v2 | https://data.jgi.doe.gov/refine-download/phytozome?organism=Spolyrhiza&expanded=290 | 92.7 | 14,924 | ATDW01000000 |
| Flax (*Linum usitatissimum*) | Linum usitatissimum v1.0 | https://data.jgi.doe.gov/refine-download/phytozome?organism=Lusitatissimum&expanded=200 | 97.7 | 20,125 | AH009791 |
| Grape (*Vitis vinifera*) | Vitis vinifera v2.1 | https://data.jgi.doe.gov/refine-download/phytozome?organism=Vvinifera&expanded=457 | 97.7 | 102,674 | NM_001280958 |
| Mouse-ear cress (*Arabidopsis thaliana*) | Arabidopsis thaliana TAIR10 | https://data.jgi.doe.gov/refine-download/phytozome?organism=Athaliana&expanded=167 | 99 | 10,898,021 | NC_003070 |
| Oil-free camphor (*Amborella trichopoda*) | Amborella trichopoda v1.0 | https://data.jgi.doe.gov/refine-download/phytozome?organism=Atrichopoda&expanded=291 | 94.4 | 29,332 | NW_006498952 |
| Oilseed rape (*Brassica napus*) | Brassica napus (AST_PRJEB5043_v1) | https://ftp.ensemblgenomes.ebi.ac.uk/pub/plants/release-55/fasta/brassica_napus/dna/ | 95.6 | 680,862 | AP018473 |
| Peach (*Prunus persica*) | Prunus persica v2.1 | https://data.jgi.doe.gov/refine-download/phytozome?organism=Ppersica&expanded=298 | 99 | 255,416 | CM007651 |
| Red clover (*Trifolium pratense*) | Trifolium pratense v2 | https://data.jgi.doe.gov/refine-download/phytozome?organism=Tpratense&expanded=385 | 93.4 | 4,571 | ASHM01022823 |
| Rice (*Oryza sativa*) | Oryza sativa v7.0 | https://data.jgi.doe.gov/refine-download/phytozome?organism=Osativa&expanded=323 | 95 | 7,711,345 | BA000010 |
| Setaria (*Setaria viridis*) | Setaria viridis v2.1 | https://data.jgi.doe.gov/refine-download/phytozome?q=%28Setaria+viridis&expanded=Phytozome-500 | 98 | 11,217,222 | CM016552 |
| Sesame (*Sesamum indicum*) | S_indicum_v1.0 | https://ftp.ensemblgenomes.ebi.ac.uk/pub/plants/release-55/fasta/sesamum_indicum/dna/ | 98.8 | 2,060,396 | XM_020696983 |
| Silvergrass  (*Miscanthus sinensis*) | Miscanthus sinensis v7.1 | https://data.jgi.doe.gov/refine-download/phytozome?organism=Msinensis&expanded=497 | 97.7 | 33,106 | AF137311 |
| Sorghum (*Sorghum bicolor*) | Sorghum bicolor v3.1.1 | https://data.jgi.doe.gov/refine-download/phytozome?q=Sorghum+bicolor | 98.3 | 1,310,030 | AF527808 |
| Sunflower (*Helianthus annus*) | Helianthus annuus r1.2 | https://data.jgi.doe.gov/refine-download/phytozome?organism=Hannuus&expanded=494 | 98 | 414,085 | AM050587 |
| Sugarcane （*R570*） | R570 | https://sugarcane-genome. cirad .fr/ | None | 21,112 | AE009947 |
| Sugarcane (*Saccharum.spontaneum*) | AP85-441 | http://sugarcane.zhangjisenlab.cn/sgd/html/download.html | 98.8 | 45,023 | MT721155 |
| Sugarcane (*Saccharum officinarum* ) | LA-purple | http://sugarcane.zhangjisenlab.cn/sgd/html/download.html | 99.2 | 67,332 | AH001756 |
| Sugarcane (*SP80-3280*) | SP80-3280 | https://figshare.com/search?q=sp80-3280 | 89.5 | 8,433 | AE009947 |
| Tomato (*Solanum lycopersicum*) | Solanum lycopersicum ITAG4.0 | https://data.jgi.doe.gov/refine-download/phytozome?organism=Slycopersicum&expanded=691 | 95.4 | 6,007,830 | LEU68072 |

^a^ The contig N50 is the length of the contig that reaches 50% of the genome size (length of all contigs) when the contigs are arranged in descending order. The larger the N50, the higher the quality of the genome assembly.

**Supplementary Table 2** The information on transcriptome sequencing data

| **Disease Type** | **ENA project number** | **Sampling site** | **Sequencing Platforms** | **Experimental materials** | **Operation** | **Operation number** | **Run** |
| --- | --- | --- | --- | --- | --- | --- | --- |
| *Surgarcane Smut* | PRJNA395694 | Leaf | Illumina HiSeq 2000 | ROC22 (Susceptible plant） | CK | R0 | SRR5922847 |
|  |  |  |  |  | 24h | R24 | SRR5909138 |
|  |  |  |  |  | 48h | R48 | SRR5922836 |
|  |  |  |  |  | 120h | R120 | SRR5922839 |
|  |  |  |  | YaCheng 05-179 (Resistant plant) | CK | Y0 | SRR5922840 |
|  |  |  |  |  | 24h | Y24 | SRR5922841 |
|  |  |  |  |  | 48h | Y48 | SRR5922843 |
|  |  |  |  |  | 120h | Y120 | SRR5922846 |
| *Ratoon stunting disease* | PRJNA507678 | Stem | Illumina Hiseq 4000 | GT11 (Susceptible plant） | CK | ST1 | SRR8269478 |
|  |  |  |  |  | 24h | ST2 | SRR8269479 |
|  |  |  |  |  | 36h | ST3 | SRR8269480 |
|  |  |  |  |  | 48h | ST4 | SRR8269475 |
|  |  |  |  |  | 120h | ST5 | SRR8269476 |
|  |  |  |  | CP72-2086 (Resistant plant) | CK | RT1 | SRR8269481 |
|  |  |  |  |  | 24h | RT2 | SRR8269482 |
|  |  |  |  |  | 36h | RT3 | SRR8269483 |
|  |  |  |  |  | 48h | RT4 | SRR8269484 |
|  |  |  |  |  | 120h | RT5 | SRR8269477 |
| *Leaf scald* | PRJNA549590 | Leaf | Illumina NovaSeq 6000 | ROC20 (Susceptible plant） | CK(1) | R0_Xa_1 | SRR9326147 |
|  |  |  |  |  | CK(2) | R0_Xa_2 | SRR9326148 |
|  |  |  |  |  | CK(3) | R0_Xa_3 | SRR9326145 |
|  |  |  |  |  | 24h(1) | R24_Xa_1 | SRR9326146 |
|  |  |  |  |  | 24h(2) | R24_Xa_2 | SRR9326143 |
|  |  |  |  |  | 24h(3) | R24_Xa_3 | SRR9326144 |
|  |  |  |  |  | 48h(1) | R48_Xa_1 | SRR9326141 |
|  |  |  |  |  | 48h(2) | R48_Xa_2 | SRR9326142 |
|  |  |  |  |  | 48h(3) | R48_Xa_3 | SRR9326139 |
|  |  |  |  |  | 72h(1) | R72_Xa_1 | SRR9326140 |
|  |  |  |  |  | 72h(2) | R72_Xa_2 | SRR9326135 |
|  |  |  |  |  | 72h(3) | R72_Xa_3 | SRR9326136 |
|  |  |  |  | LCP 85-384 (Resistant plant) | CK(1) | S0_CK_1 | SRR9326133 |
|  |  |  |  |  | CK(2) | S0_CK_2 | SRR9326134 |
|  |  |  |  |  | CK(3) | S0_CK_3 | SRR9326131 |
|  |  |  |  |  | 24h(1) | S24_Xa_1 | SRR9326132 |
|  |  |  |  |  | 24h(2) | S24_Xa_2 | SRR9326129 |
|  |  |  |  |  | 24h(3) | S24_Xa_3 | SRR9326130 |
|  |  |  |  |  | 48h(1) | S48_Xa_1 | SRR9326137 |
|  |  |  |  |  | 48h(2) | S48_Xa_2 | SRR9326138 |
|  |  |  |  |  | 48h(3) | S48_Xa_3 | SRR9326152 |
|  |  |  |  |  | 72h(1) | S72_Xa_1 | SRR9326151 |
|  |  |  |  |  | 72h(2) | S72_Xa_2 | SRR9326150 |
|  |  |  |  |  | 72h(3) | S72_Xa_3 | SRR9326149 |
| *Mosaic virus disease* | PRJNA379719 | Leaf | Illumina HiSeq 2000 | ROC22 (Susceptible plant） | Healthy leaves | T1 | SRR5980284 |
|  |  |  |  |  | ENA Data | T2 | SRR5980285 |
|  |  |  |  |  | Diseased leaves | T3 | SRR5980283 |

**Supplementary Table 3** The results of transcriptome data quality control

| **Sra name** | **^a^Raw bases**  **(G)** | **^b^Clean bases**  **(G)** | **^c^Q20 bases**  **(%)** | **^d^Q30 bases**  **(%)** | **^e^GC_content**  **(%)** |
| --- | --- | --- | --- | --- | --- |
| SRR5909138 | 5.77 | 5.63 | 95.88 | 87.11 | 53.72 |
| SRR5922836 | 6.7 | 6.54 | 95.87 | 87.07 | 53.68 |
| SRR5922839 | 5.47 | 5.34 | 95.88 | 87.12 | 53.67 |
| SRR5922840 | 5.99 | 5.88 | 96.18 | 87.73 | 53.78 |
| SRR5922841 | 5.27 | 5.18 | 96.23 | 87.77 | 54.17 |
| SRR5922843 | 6.18 | 6.07 | 96.17 | 87.65 | 54.35 |
| SRR5922846 | 5.55 | 5.45 | 96.2 | 87.71 | 54.32 |
| SRR5922847 | 8.27 | 7.99 | 95.9 | 88.19 | 53.65 |
| SRR8269475 | 7.9 | 7.61 | 97.29 | 93.02 | 56.3 |
| SRR8269476 | 8.33 | 8.04 | 97.33 | 93.07 | 54.51 |
| SRR8269477 | 8.73 | 8.39 | 97.18 | 92.75 | 54.61 |
| SRR8269478 | 8.48 | 8.15 | 97.26 | 92.94 | 55.33 |
| SRR8269479 | 12.22 | 11.81 | 97.38 | 93.21 | 55.35 |
| SRR8269480 | 7.64 | 7.37 | 97.32 | 93.07 | 55.43 |
| SRR8269481 | 7.51 | 7.24 | 97.31 | 93.05 | 55.35 |
| SRR8269482 | 10.27 | 9.91 | 97.35 | 93.13 | 56.1 |
| SRR8269483 | 9.03 | 8.71 | 97.31 | 93.06 | 56.29 |
| SRR8269484 | 12.32 | 11.89 | 97.34 | 93.12 | 55.37 |
| SRR9326129 | 7.16 | 7.11 | 98.13 | 94.46 | 57.91 |
| SRR9326130 | 6.63 | 6.58 | 98.22 | 94.9 | 57.62 |
| SRR9326131 | 7.95 | 7.9 | 97.68 | 93.37 | 58.94 |
| SRR9326132 | 7.41 | 7.35 | 98.15 | 94.74 | 57.86 |
| SRR9326133 | 8.19 | 8.13 | 98.22 | 94.75 | 59.45 |
| SRR9326134 | 7.01 | 6.96 | 97.66 | 93.31 | 58.6 |
| SRR9326135 | 6.4 | 6.36 | 98.13 | 94.41 | 57.09 |
| SRR9326136 | 10.67 | 10.59 | 97.88 | 93.93 | 56.88 |
| SRR9326137 | 6.94 | 6.89 | 98.16 | 94.74 | 56.87 |
| SRR9326138 | 6.14 | 6.09 | 98.15 | 94.72 | 57.22 |
| SRR9326139 | 6.63 | 6.57 | 97.45 | 93.07 | 56.85 |
| SRR9326140 | 7.35 | 7.3 | 97.72 | 93.69 | 57.24 |
| SRR9326141 | 7.24 | 7.19 | 97.61 | 93.16 | 56.21 |
| SRR9326142 | 7.77 | 7.7 | 97.33 | 92.74 | 56.06 |
| SRR9326143 | 7.13 | 7.09 | 97.75 | 93.52 | 57.09 |
| SRR9326144 | 7.88 | 7.82 | 97.56 | 93.08 | 57.03 |
| SRR9326145 | 9.01 | 8.94 | 97.67 | 93.31 | 57.53 |
| SRR9326146 | 8.06 | 8 | 97.34 | 92.53 | 56.65 |
| SRR9326147 | 7.06 | 7.01 | 97.74 | 93.68 | 57.32 |
| SRR9326148 | 7.63 | 7.57 | 97.57 | 93.07 | 57.34 |
| SRR9326149 | 8.75 | 8.69 | 98.12 | 94.68 | 57.96 |
| SRR9326150 | 6.73 | 6.68 | 98.1 | 94.61 | 58.03 |
| SRR9326151 | 7.25 | 7.19 | 97.99 | 94.35 | 57.8 |
| SRR9326152 | 7.47 | 7.43 | 98.08 | 94.32 | 57.09 |
| SRR5980283 | 7.34 | 5.85 | 96.99 | 91.64 | 56.34 |
| SRR5980284 | 4.68 | 3.73 | 96.91 | 91.46 | 56.98 |
| SRR5980285 | 6.02 | 4.88 | 97.02 | 91.69 | 56.24 |

^a^ Raw data yield

^b^ Amount of valid data after quality control

^c^ The mass value ≧ 20 bases as a percentage of all bases and the error rate of 20 is 1%.

^d^ The mass value ≧ 30 bases as a percentage of all bases and the error rate of 30 is 0.1%.

^e^ Number of guanine and cytosine in clean bases as a percentage
